# Terpenes from essential oils and hydrolate of *Teucrium alopecurus* triggered apoptotic events dependent on caspases activation and PARP cleavage in human colon cancer cells through decreased protein expressions

**DOI:** 10.18632/oncotarget.25955

**Published:** 2018-08-17

**Authors:** Fatma Guesmi, Amit K. Tyagi, Sahdeo Prasad, Ahmed Landoulsi

**Affiliations:** ^1^ Department of Experimental Therapeutics, University of Texas MD Anderson Cancer Center, Houston, TX, USA; ^2^ Laboratory of Biochemistry and Molecular Biology, Faculty of Sciences of Bizerte, University of Carthage, Tunis, Tunisia

**Keywords:** Teucrium alopecurus, oily fractions, water soluble fractions, human colon cancer cells, gene expression

## Abstract

This study focused on characterizing the Hydrophobic and Hydrophilic fractions of *Teucrium alopecurus* in the context of cancer prevention and therapy. The goal was also to elucidate the molecular mechanisms involved and to determine its efficacy against cancer by triggering apoptosis and suppressing tumorigenesis in human colon cancer. The data here clearly demonstrated that oily fractions of *Teucrium alopecurus* act as free radical scavengers, antibacterial agent and inhibited the proliferation of HCT-116, U266, SCC4, Panc28, KBM5, and MCF-7 cells in a time- and concentration-dependent manner. The results of live/dead and colony formation assays further revealed that *Teucrium* essential oil has the efficacy to suppress the growth of colon carcinoma cells. In addition, essential oil of *Teucrium alopecurus* induced apoptosis, as indicated by cleavage of caspases-3, -8, and -9 and poly-adenosine diphosphate ribose polymerase. Moreover, *Teucrium alopecurus* essential oil suppressed gene expression involved in survival, proliferation, invasion, angiogenesis, and metastasis in human colon cancer cells. No sign of toxicity was detected *in vivo* after treatment with increasing concentrations of essential oil. Oral administration of T.alopecurus inhibited LPS-induced colon inflammation. This anticancer property of this specie *Teucrium alopecurus* fractions could be due to their phenolic and/or sesquiterpene content (d-limonene, α-Bisabolol, Humulene, Thymol, and (+)-epi-Bicyclosesquiphellandrene). Hence our study reveals the anticancer activity of *Teucrium alopecurus* oil mediated through the suppression of cell growth, cell proliferation, and the induction of apoptosis of cancer cells. Thus, it has potential to be developed as an anticancer agent; however more *in vitro* and *in vivo* studies are warranted.

## INTRODUCTION

Colonic carcinoma, a multistage process that occurs over many years and is regulated by multiple signaling pathways, is the target of numerous anticancer therapies [1]. This disease involves many proteins until the first step of the tumor to the ultimate stade. The combination of chemotherapeutic drugs with terpenes could be of great clinical importance in cancer therapy [2]. Colorectal cancer (CRC), one of the most lethal cancers, especially when it reaches advanced stages [3] and develops resistance to chemotherapeutic agents over time [4], evolves through a multistep process in which normal mucosa is first transformed into adenomatous polyps and then eventually into invasive carcinoma [5]. Colon carcinogenesis progresses over many years via molecular events by the modification of genetic sequences, called as the adenoma—carcinoma sequence [6]. Genetic alterations in somatic cells (mutations) include the up-regulation of proto-oncogenes and the down-regulation of tumor suppressor genes [7].

Using essential oils (EOs) from aromatic plants is one traditional alternative method that has been reported to have anticancer properties [8, 9], both *in vitro* and *in vivo* [10], against mouth, breast, lung, prostate, liver, colon, and brain cancer and even leukemia [11–16]. Numerous nutraceuticals from “mother nature” could be potential treatments for CRC [5]. These nutraceuticals target various steps in tumor cell development [1] and have been shown to potentially halt cancer progression by targeting one or more steps in the cell cycle [5]. Many researchers have demonstrated the anticancer effect of essential oils [17–21]. The chemical composition of essential oils can act as an anti-inflammatory, affecting arachidonic metabolism or cytokine production or the modulation of pro-inflammatory gene expression [22]. Natural products such as terpenes, a class of molecules characterized by the presence of multiple terpenic groups in their structural moiety, have emerged as alternatives to treat a broad range of human diseases, including particularly cancer and inflammation [23]. The whole botanical may be better than its active principle [5].

The Teucrium (Lamiaceae) genus contains many species that are distributed mainly in the Mediterranean basin [24]. Phenolic and terpenic components extracted from Teucrium species possess the ability to treat obesity, hypercholesterolemia, and diabetes, as well as antiinflammatory, antimicrobial, and anticancer properties [25]. *T. polium* protects liver cells against hepatocellular carcinoma in carcinogenesis-induced animal models [26]. It has been shown to be an effective and safe chemosensitizer agent for cancer therapy [25].

This report describes novel insight into the curative effect of hydrophobic fraction of Teucrium on cancer. *Teucrium alopecurus* (H'chichit ben salem), widely used in traditional medicine, is known to possess anti-inflammatory properties. The chemical investigation of the aerial parts has yielded bioactive compounds. Earlier studies showed that some of these compounds inhibit the proliferation of tumor cells. Our goal in this report was to investigate the possible use of essential oil (TA-1) and hydrolate (TA-2) of *Teucrium alopecurus* as an alternative complementary cancer treatment, and, in order to elucidate its potential activity and the mechanisms underlying these effects, this species was tested on colorectal carcinogenesis *in vitro*.

## RESULTS

### Analysis of hydrophobic and hydrophilic fractions with gas chromatography-mass spectrometry (GC/MS)

**Figure 1:**
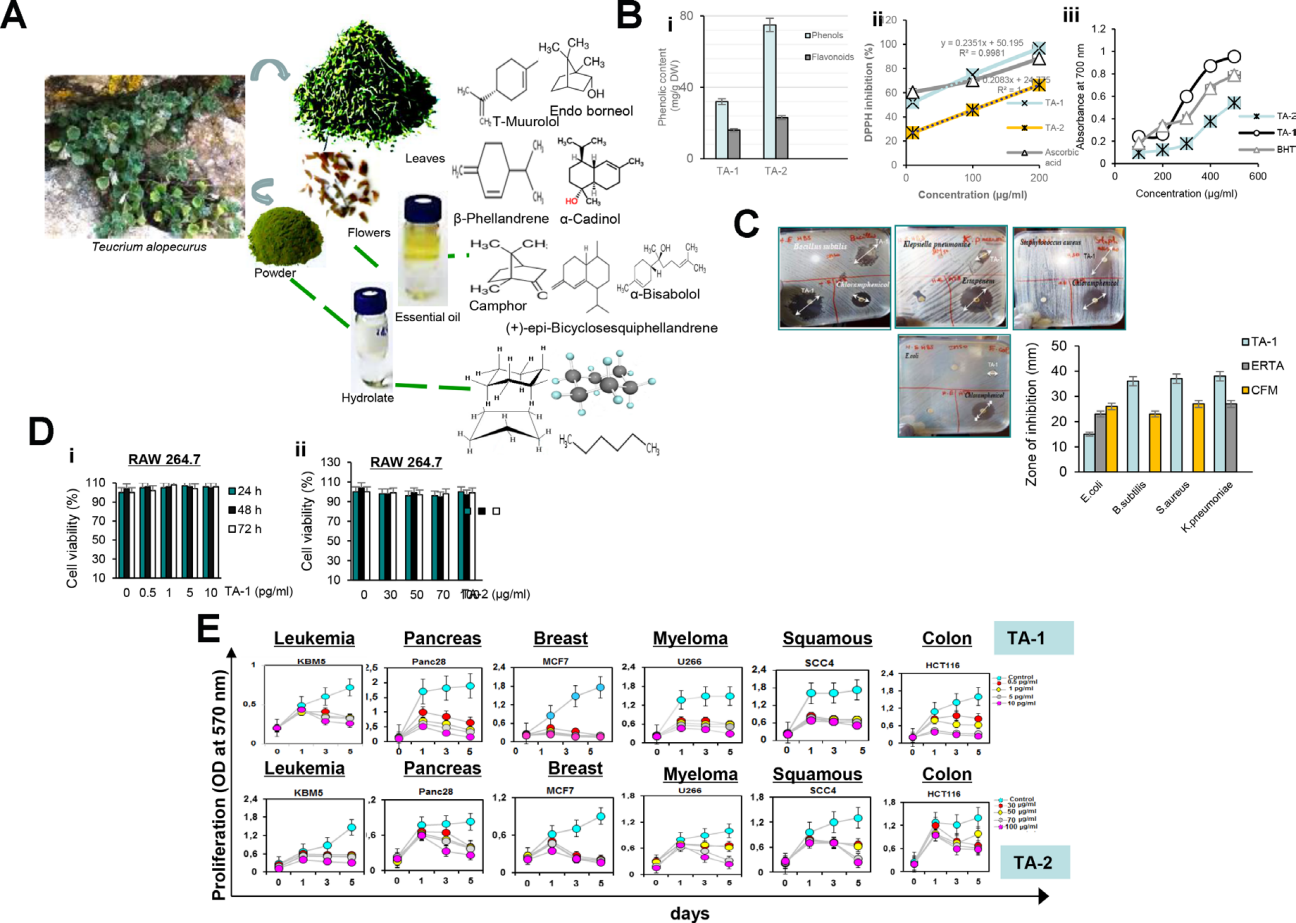
(**A**) Chemical structures of the major compounds of Hydrophobic (TA-1) (i) and Hydrophilic (TA-2) (ii) fractions of *Teucrium alopecurus*; (**B**) Phenolic content (i) and antiradical potential of TA-1 and TA-2 using DPPH (ii) and FRAP (iii) assay; (**C**) Antibacterial activity of hydrophobic fraction of *Teucrium alopecurus*. Plant essential oil (1 μl) were applied per disc (Whatman No 5 mm) onto the seeded top layer of the agar plates containing tested bacteria (*E.coli*, *S.aureus*, *B.subtilis* and *K.pneumoniae*). Essential oil was tested with a Chloramphenicol, and Ertapenem discs as positive controls. DMSO discs were used as negative controls. The plates were incubated at 37°C for 24 h, and zone of inhibition was determined. (**D**) Effect of TA-1 and TA-2 on RAW cell viability. RAW 264.7 cells, seeded in triplicate in 96-well plates, were pre-treated with the concentrations of TA-1 (0.5, 1, 5, 10 pg/mL) (i) and TA-2 (30, 50, 70, 100 μg/mL) (ii) and incubated for 24 h, 48 h and 72 h. Cell viability was analyzed with the MTT assay. (**E**) Effect of TA-1 and TA-2 on tumor cell proliferation. KBM-5, HCT-116, Panc-28, MCF-7, U266, and SCC4 cells (5 × 10^3^ cells/well) were seeded in triplicate in 96-well plates, treated with different concentrations of TA-1 (0.5, 1, 5, 10 pg/mL) and TA-2 (30, 50, 70, 100 μg/mL); cell growth was analyzed on days 0, 1, 3, and 5 by tetrazolium salt 3-(4-5-dimethylthiozol-2-yl)2-5-diphenyl-tetrazolium bromide (MTT) assay. The results show the mean ± SD values. These are representative results of three independent experiments.

The essential oils and hydrolats of the aerial parts of *Teucrium alopecurus* were analysed qualitatively and quantitatively. Forty-eight compounds were identified and listed in Supplementary Table 1 and Supplementary Figure 1. Essential oil from *Teucrium alopecurus* showed that sesquiterpenes are the most abundant skeletons. Figure 1Ai shows that TA-1 is mainly composed of (+)-epi-Bicyclo sesquiphellandrene, α-Bisabolol, T-Muurolol, α-Cadinol, β- Phellandrene, and d-limonene (Figure 1(Ai)). Of these terpene compounds, the most abundant was α-Bisabolol (16.16%). However, organic compounds were the only components of TA-2 (Figure 1(Aii)). It was noted that a small amount of essential oil was dissolved in the hydrosol. Distillation with a Clevenger apparatus completely extracted the essential oils and led to no loss of volatile molecules from *Teucrium alopecurus*.

### Phenolic contents

As indicated in Figure 1Bi, phytochemical study indicated an important level of phenolic contents, including total phenols and Flavonoids.

### TA-1 and TA-2 scavenge free radicals

It has been reported in Figure 1Bii and Figure 1Biii that *Teucrium* specie is indicated as potent free radical scavengers of the DPPH radicals and can also reduce the Fe^3+^/ferricyanide complex to the ferrous form, the antioxidant effect is close to that of the standard ascorbic acid and BHT.

### Antibacterial activity of TA-1

As reported in Figure 1C, essential oil isolated from *Teucrium alopecurus* was more effective (*P* < 0.05) in inhibiting all tested bacteria, than those of Chloramphenicol (10 μg/μl) (CFM) and Ertapenem (10 μg/μl) (ERTA).

### Cell viability of RAW 264.7 macrophage

As shown in Figure 1D, MTT assay did not show any significant difference (*P* > 0.05) in RAW 264.7 cell viability among the control and TA-1 or TA-2-treated groups, this indicated that *Teucrium alopecurus* is not cytotoxic.

### TA-1 and hydrophilic fraction (TA-2) represses the proliferation of colorectal cancer cells

Figure 1E shows the concentration- and time-dependent repression of tumour cell proliferation induced by *Teucrium*. Breast cancer cells (MCF-7) and pancreatic carcinoma (Panc28) cells are the most sensitive to TA-1 at 5 and 10 pg/mL between three and five days and present low cell growth compared to the vehicle control (Figure 1E, upper panel). Chronic myelogenous leukemia (KBM-5) and human colon cancer cells (HCT116) cells have a moderate sensitivity to this essential oil. In addition, TA-2 suppresses the proliferation of cancer cells (Figure 1D, lower panel).

### Teucrium fractions decrease clonogenic potential

**Figure 2:**
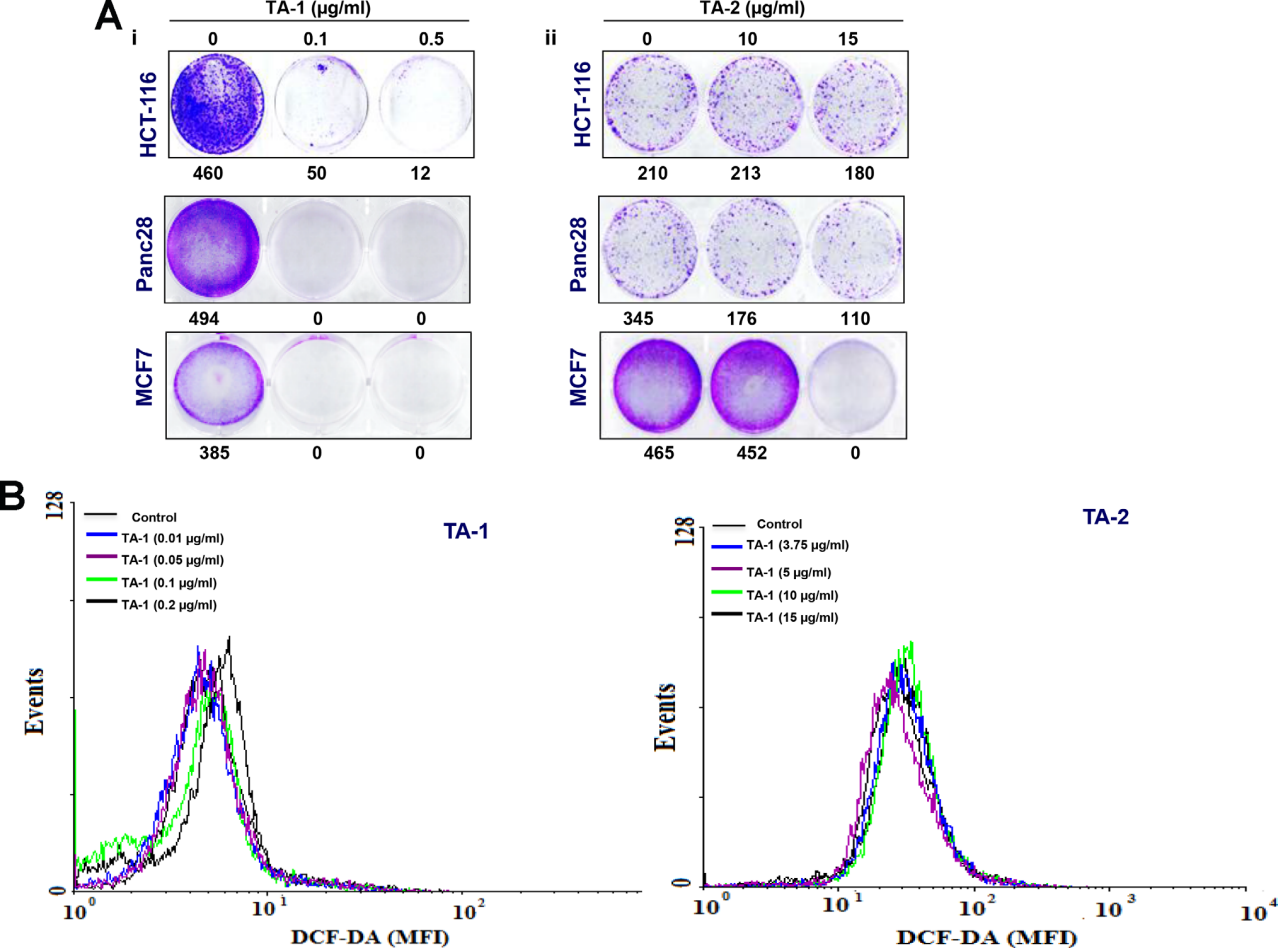
(**A**) Effects of TA-1 and TA-2 on the clonogenic potential of tumor cells; cells were plated in six-well plates in FBS-containing medium, then exposed to different concentrations of TA-1 and TA-2. At this point, the drug treatments were washed out and replaced with fresh medium. The ability of individual cells to form colonies was assessed 9 days posttreatment. (**B**) TA-1 induces ROS production. HCT-116 (1 × 10^6^ cells) were labeled with dichlorodihydrofluorescein diacetate (DCF-DA), treated with the concentrations of TA-1 and TA-2 indicated for 1 h, and then examined for ROS production by flow cytometer. ^*^ is the significance of difference compared with vehicle control; *p* < 0.05. These are representative results of three independent experiments.

Treatment with hydrophobic and hydrophilic fractions of Teucrium suppresses the clonogenic potential of colon cancer cells. HCT-116 cell lines were very resistant to low and high concentrations of TA-2, showing only ∼5% reduction in clonogenic potential (Figure 2(Ai)). However, treatment with a high concentration of TA-1 caused nearly 100% inhibition (Figure 2(Aii)). KBM5 cells were very sensitive to both high concentrations of TA-2 and various concentrations of TA-1.

### *Teucrium* induces radical oxygen species (ROS) generation

The oily fraction of *Teucrium alopecurus* was able to induce ROS generation (Figure 2B). A significant (*p* < 0.05) increase in ROS levels at higher doses of 0.1 μg/mL (MFI 125.4) and 0.2 μg/mL (MFI 142.4) TA-1 compared to the control (MFI 114.2) was observed in colon cancer cells (Left panel). However, very low amounts (non-significant) of ROS were produced in HCT-116 treated with hydrophilic fractions of teucrium (Right panel).

### Live/dead assay

**Figure 3:**
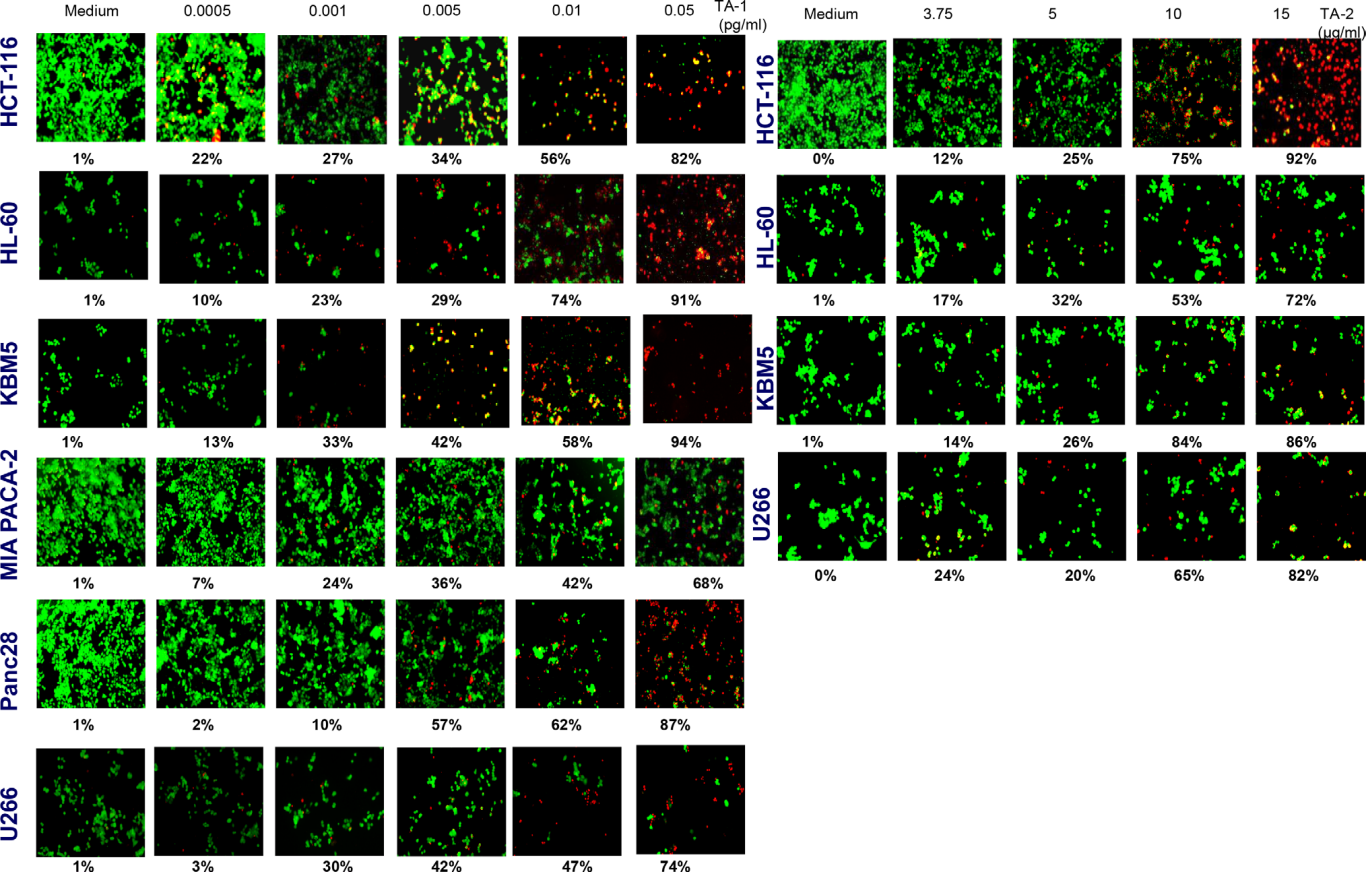
Tumor cells were treated with different concentrations of TA-1 and TA-2 Cells were stained with the Live/Dead reagent (5 mM ethidium homodimer and 5 mM calcein AM) and incubated at 37°C for 30 min. Cells were analyzed under a fluorescence microscope (Labophot-2; Nikon, Tokyo, Japan). Percentages are proportions of apoptotic cancer cells. The results are representative of three independent experiments. Viable cells are green and non-viable cells are red. Magnification = 20×.

The treatment of various tumor cell cultures with TA-1 volatile oil and TA-2 hydrolate for 48 h induces a concentration-dependent decreased cell number, but each cell line showed different sensitivity (Figure 3). Exposing different tumor cells to various concentrations of oily (Left panel) and soluble fractions (Right panel) of teucrium induced cell death in a concentration-dependent manner and significantly increased the number of apoptotic cells from 1% to ∼100% and from 1% to 80%, respectively.

### Caspases activation and poly-adenosine diphosphate ribose polymerase (PARP) cleavage

**Figure 4:**
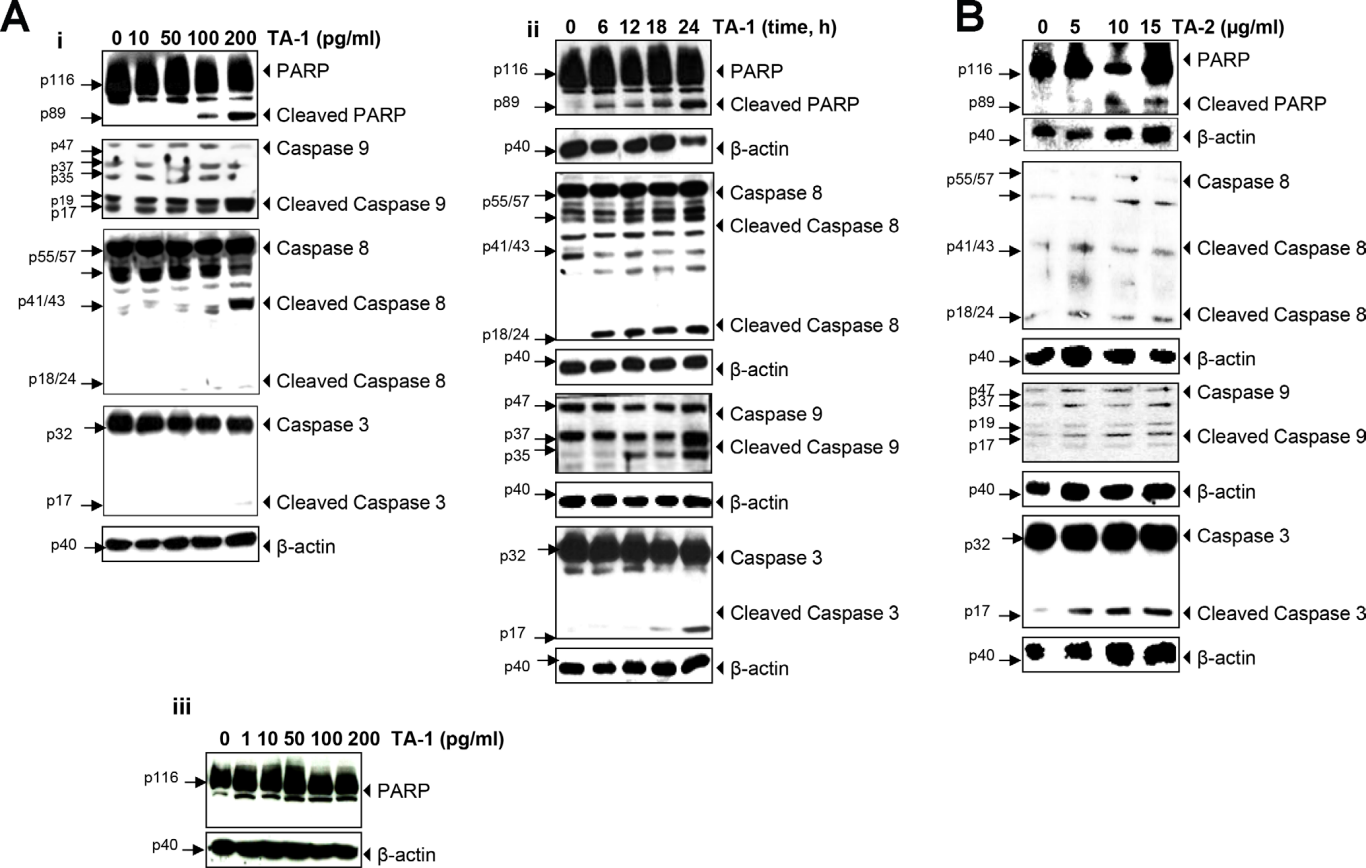
TA-1 and TA-2 induces caspase activation and poly-adenosine diphosphate ribose polymerase (PARP) cleavage in HCT-116 cells in a concentration- and time-dependent manner (**A**) Whole-cell extracts of HCT-116 and HUVECs cells were treated with different concentrations of TA-1 and then analyzed by Western blotting using the antibodies indicated and (**B**) HCT-116 Cells were treated with 200 pg/mL TA-1 for the indicated times, and whole cell extracts were prepared and analyzed using the indicated antibodies. These are representative results of three independent experiments.

The different times and concentrations when exposing HCT-116 cells to teucrium essential oil indicate that this essence protects the cells from DNA degradation. The results above show that Teucrium induced significant caspase activation and PARP cleavage in a concentration- and time-dependent manner (Figure 4 (Ai–Aii) and 4B), thus indicating that colorectal cancer cells are undergoing apoptosis.

As shown in Figure 4Aiii, *Teucrium alopecurus* did not induced PARP cleavage in human umbilical vein endothelial cells (HUVECs), this indicated that essential oil is not cytotoxic to normal cells.

### TA-1 and TA-2 suppress the expression of gene products involved in the antiapoptotic, proliferation, metastasis, invasion and angiogenesis of colorectal cancer cells

**Figure 5:**
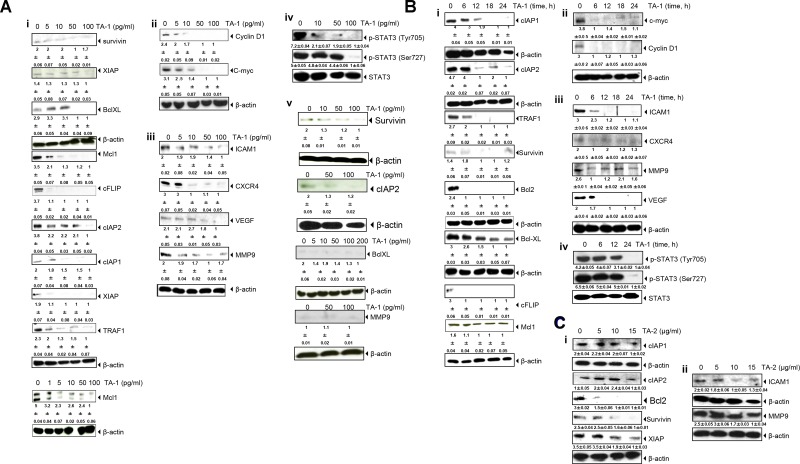
TA-1 suppresses (**A**i, **B**i) antiapoptotic, (**A**ii, **A**iii, **B**ii, **B**iii) proliferative, and metastatic gene products and (**A**iv, **B**iv) inhibits STAT3 phosphorylation in HCT-116 cell lines in a time- and concentration-dependent manner. TA-1 had no effect on HUVECs cells (Aiii). TA-2 suppresses (**C**i) antiapoptotic and (**C**ii) metastatic gene products. The cells were treated with the concentrations of TA-1or TA-2 indicated for 24 h. Whole cell protein extracts were prepared, separated by electrophoresis, and then transferred to the nitrocellulose membrane using the antibodies indicated. These are representative results of three independent experiments.

The down-regulation of multiple gene products obtained from the western blot analysis of the TA-1 effect explains a molecular mechanism of blocking gene expression in response to teucrium essential oil treatment in a concentration-dependent (Figure 5 (Ai–Aiii)) and time-dependent (Figure 5 (Bi–Biii)) manner. As shown in Figure 5Av, no expression of gene products in HUVECs cells was detected after treatment with TA-1. Western blotting and relative densitometric analyses of the suppression of antiapoptotic, proliferative, and metastatic gene products and the inhibition of Signal transducer (STAT3) phosphorylation cellular levels by TA-1 essential oil in HCT-116 cell lines at different concentrations and time points are shown in Supplementary Figure 2. Blots have been done three times.

**Figure 6:**
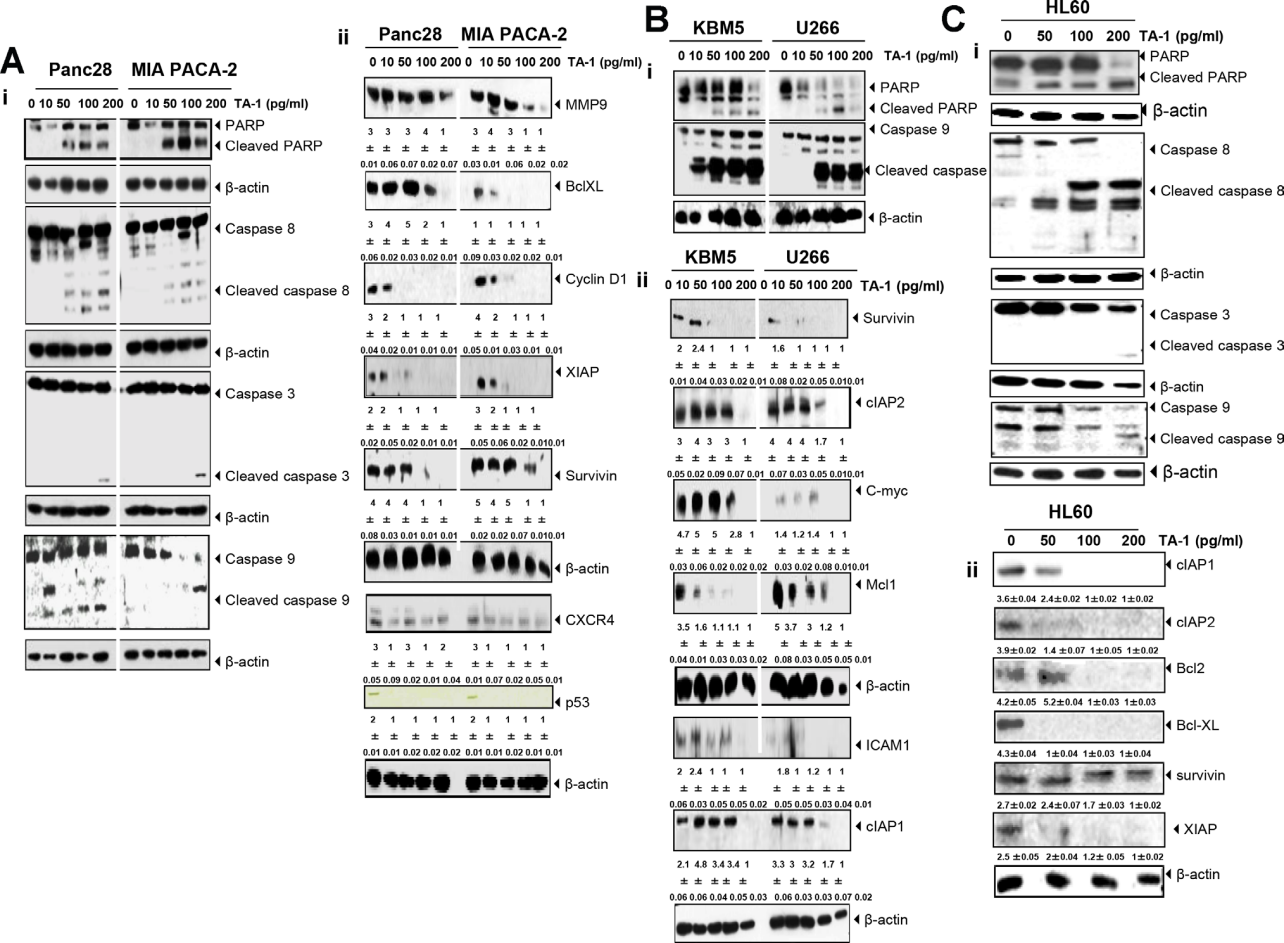
TA-1 induces PARP cleavage and caspases activation and suppresses the expression of gene products involved in tumor cell survival, proliferation, and metastasis in (**A**) Panc28- and MIA PACA-2 cells, (**B**) KBM5 and U266 cells, (**C**) and HL60 cells. The cells were treated with the concentrations of TA-1 indicated for 24 h. Whole cell protein extracts were prepared, separated by electrophoresis, and then transferred to the nitrocellulose membrane using the antibodies indicated. These are representative results of three independent experiments.

As shown in Figure 5 (Bi, Bii) the c-*myc* as cell cycle components and the cell cycle regulator protein (cyclin D1)were down-expressed within 6 h of teucrium essential oil treatment, whereas Intercellular adhesion molecule-1 (ICAM1), an inhibitor of apoptosis protein (IAP)-1, IAP-2 proteins were not down-regulated, even after 12 h of treatment in HCT-116 cells. Complete abrogation of most gene expression appeared upon treatment with 100 pg/mL TA-1.

There was a low concentration-dependent decrease in the expression of various genes upon hydrosol treatment, with the maximal inhibition effect observed at 15 μg/mL (Figure 5 (Ci, Cii)).

### TA-1 suppresses constitutive STAT3 activation in HCT-116 cells

Colorectal cancer cells express constitutively active STAT3 [27]. Upon exposing tumor cells to various concentrations of essential oils, prepared cell lysates were investigated for the phosphorylation of STAT3 (Tyr^705^) and (Ser^727^) and performed by western blot analysis. Interestingly, these results show the concentration- and time-dependent repression of STAT-3 activation induced by TA-1 in HCT-116 cells (Figure 5 (Aiv, Biv)). The maximum inhibitory activity occurred at 100 pg/mL, and the optimum time for the repression of STAT3 phosphorylation was at 24 h.

### Acute cytotoxic effect of TA-1

**Figure 7:**
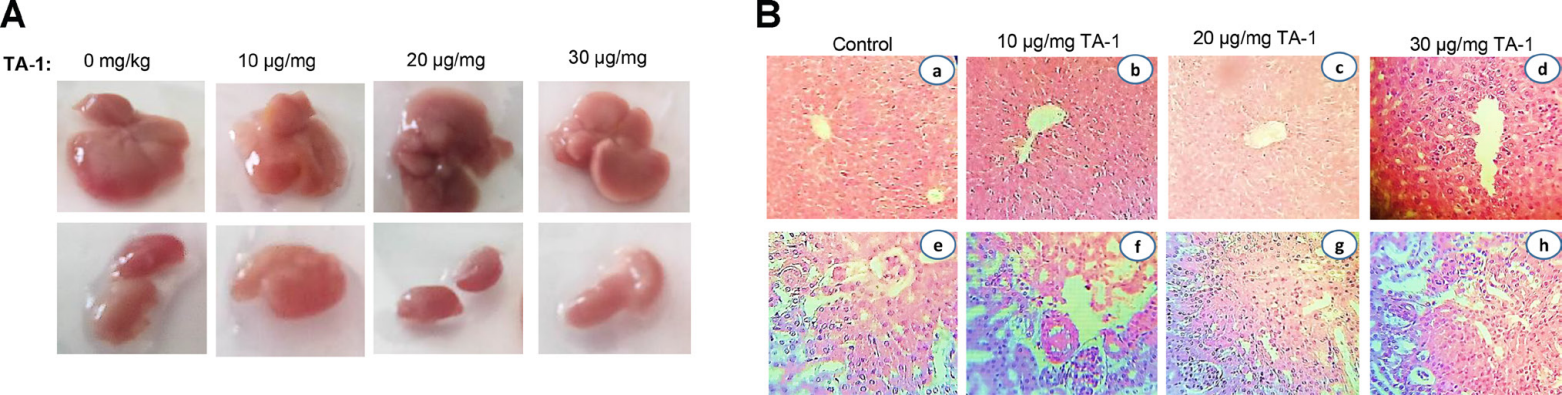
Macroscopical view of mice liver and kidney (**A**) and histological sections of liver and kidney from the acute toxicity test (**B**). No significant differences in the structure of liver and kidney between treated and control groups (Hematoxylin & Eosin stain). Magnification: a, b and c, ×10; d, e, f and h ×40. Data are represented as x ± s (*n* = 6).

Figure 7 shows the pharmacological study of the general toxicity of essential oil in mice. TA-1 at different doses of 10, 20 and 30 μg Kg^−1^ exhibited no signs of toxicity (Figure 7A). Histological overview showed no abnormal damage in liver and kidney and any difference between treated and untreated groups (Figure 7B). After 7 days of observation, there were no mortality and no behavioural changes were detected.

### Anti-inflammatory effect of TA-1

**Figure 8:**
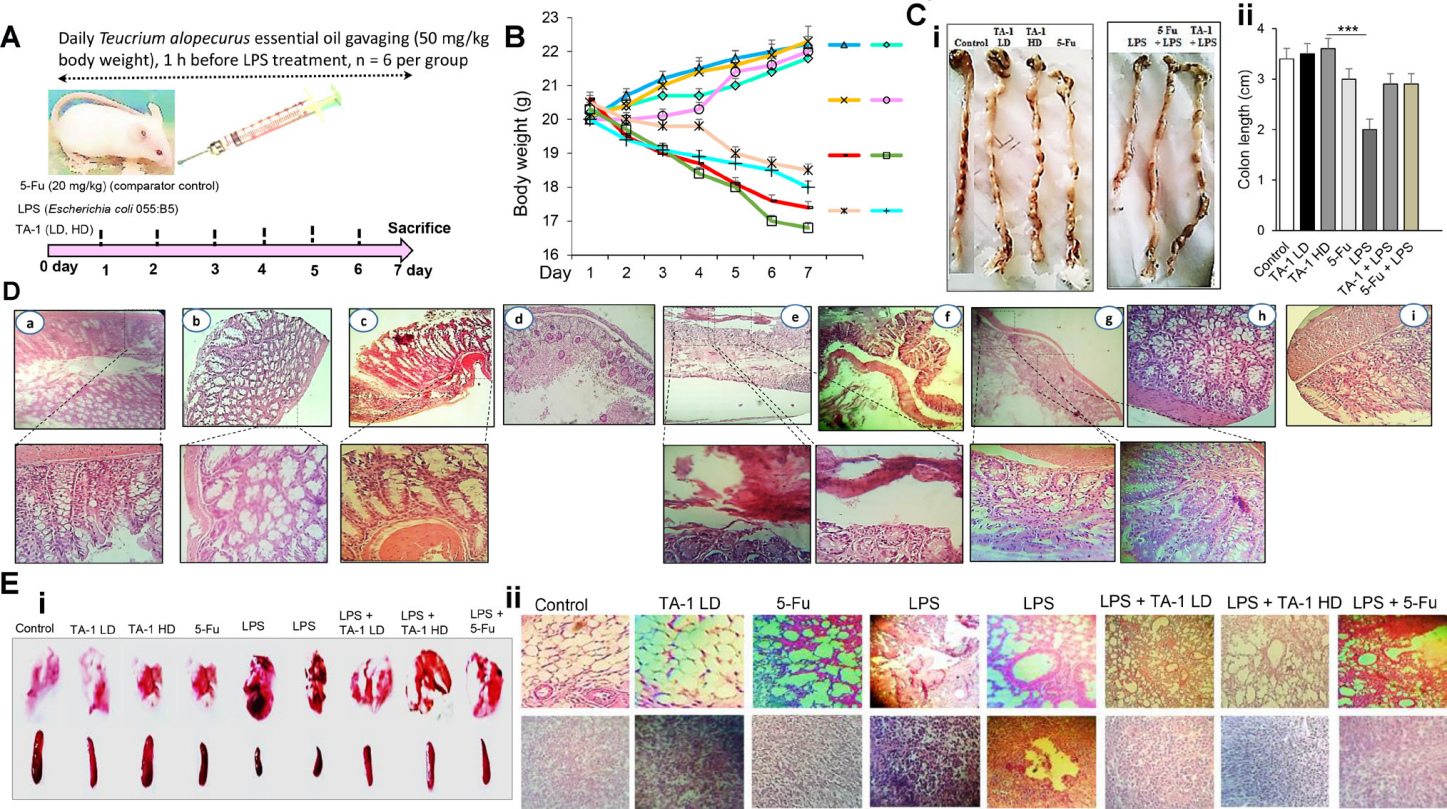
(**A**) Experimental strategy of the model with days of exposure to LPS and proposed therapy with essential oil; (**B**) animals weight during treatment; (**C**) macroscopical view of mice colon (i) and colon lenght (ii) of each group; (**D**) microscopical analysis of colon tissues of TA-1 (LD) group (a), TA-1 (HD) group (b), 5-Fu group (c), LPS group (d, e and f), LPS + TA-1 group (g, h), and LPS + 5-Fu group (i). (**E**) macroscopical view (i) and the histological overview (ii) of lung and spleen of different groups. TA-1: *Teucrium alopecurus* essential oil; LD: low dose; HD: high dose; 5-Fu: 5-Fluorouracil; LPS: Lipopolysaccharides.

As shown in Figure 8, mice were treated with TA-1 low dose (LD) and high dose (HD) 1 h before treatment with lipopolysaccharide (LPS) (*Escherichia coli* 055:B5) as inflammatory agent (Figure 8A) used to induce inflammation and cancer in colon cells. After experimental analysis, LPS was shown to induce body weight loss (Figure 8B), inhibition of colon length (Figure 8C), inflammation in colon (Figure 8D) and other parts of digestive system (Supplementary Figure 4), which have an effect on weight and color of feces. In addition, LPS induced damage in spleen and lung tissues (Figure 8E) compared with control group. Whereas, the therapeutic effect of essential oil was clearly investigated. We have found that TA-1 could effectively decrease the damage of colon, spleen and lung cells compared with comparator control group (5-Fu). The results indicated that 5-Fu could reduce the inflammation of colon cells which increased by LPS.

## DISCUSSION

The aim of this study was to determine whether Teucrium essential oil led to antitumor activity by abrogating the STAT3 signaling pathway in colon cancer cells. An increased level of apoptotic CRC cells was observed, as well as inhibited proliferation using MTT and Live/Dead assays.

Natural bioproducts are invaluable resources in drug discovery [28]. Many reports have documented the antitumor effects of essential oils and their bioactive compounds. In the literature, it has been reported that muurolol, δ-cadinene, and spathulenol [22, 29] are the most abundant bioactive compounds of essential oils extracted from the aerial parts of some species belonging to the genus Teucrium (*T. brevifolia* Schreber, *T. montbretii* Benth. ssp. *heliotropiifolium* (Barbey) Davis, *T. flavum*, and *T. capitatum*). In our work, 1.78% of δ-cadinene in TA-1 leaves was found, whereas there was a low amount of tau.-Muurolol.

Previous reports on the chemical composition of *Teucrium alopecurus* essential oil growing in Tunisia showed sesquiterpenes, mainly δ-cadinene (13.4%), α-humulene (12.3%), and nerolidyl acetate (12.3%), as its major components [30]. There is also data in the literature about the most abundant fatty acids, where Linolenic, linoleic, and palmitic acids together made up 74.2% of *Teucrium alopecurus* collected from Matmata, Gabes (Tunisia), and the highest number of sterols were stigmasterol, sitosterol, phytosterol, and clerosterol [31].

The cytotoxic effects exhibited by essential oils could be related to an overall action of the compounds present in oils, especially to sesquiterpene compounds [32, 33], phenols, alcohols, and monoterpene aldehydes [34, 35]. TA-1, with its bioactive compounds, and hydrosol elicit a cytotoxic effect by inhibiting cell viability. In the study done by El Hadri *et al.* [36], α-humulene was found to be cytotoxic against MCF-7, HCT-116, and murine macrophage (RAW264.7) cell lines. The cytotoxic potential of β-pinene was investigated in MCF-7, A375, and HepG2 cancer cells and in other different tumor and nontumor cell lines [37]. In fact, the studies of the cytotoxicity of cancer cell lines towards essential oils demonstrate a great difference between them with respect to their sensitivity to the substances contained in the oily fraction [38] of TA-1.

Various cell cycle checkpoints act as potential targets for cancer treatment [10]. Several nutraceuticals prevent cancer cells from transitioning from the G1 to S phase. EOs and their constituents serve as effective anticancer substances by targeting cell cycle progression in cancer cells [10]. In colorectal cancer cells, the maximal alteration in cancer cell cycle progression in the G2/M phase promoted by TA-1 was observed at 24 h. Volatile oils and their constituents serve as effective anticancer substances by targeting cell cycle progression in cancer cells [10]. Thymol, a phenolic compound of Teucrium essential oil, induced cell cycle arrest at the G0/G1 phase [39]. Gene products involved in cell proliferation were found here to be overexpressed in HCT116 cells. Hydrophobic and hydrophylic fractions decreased cell proliferation in human colon cancer cells through a decrease of Cyclin D1 and *p21*.

Cancer cells possess several abilities like resistance to growth inhibition, proliferation without dependence on growth factors, replication without limit, evasion of the apoptosis, and the invasion, metastasize, and support of angiogenesis [1].

TA-1 and TA-2 were found here to suppress the proliferation of various types of tumor cells. Based on the data in the literature, it is possible to hypothesize that the antiproliferative effect of oily fractions was correlated with major terpenes constituents, α-Bisabolol as a sesquiterpene alcohol, and (+)-epi-Bicyclosesquiphellandrene. In a further study on highly malignant glioma cells, Edris [20] evaluated that α-Bisabolol is considered a promising inducer of apoptosis. According to the work of Tyagi *et al.* [40], β-sesquiphellandrene was found to have antiproliferative effects comparable to those of curcumin in human leukemia, multiple myeloma, and colorectal cancer cells. d-limonene, as a constituent of TA-1 in a small amount, is able to inhibit cell proliferation and enhance apoptosis [20]. Another sesquiterpene present in TA-1, namely λ-Humulene, has been identified for its pro-apoptotic activities, mainly against colorectal carcinoma [2].

A recent report by Escandary *et al.* [41] also investigated the antiproliferative activity of *T. polium* on different cell lines. *T. polium* has been shown to be an effective and safe chemosensitizer agent for cancer therapy [25].

It is interesting to note that inducing apoptosis depends on an extrinsic and intrinsic pathway. The intrinsic pathway is mitochondria dependent, whereas the extrinsic is triggered by death receptors (DRs) [1]. PARP-1 cleavage induced by derivative compounds of EOs contributes to the change of the DNA repair process in the cancer cells [10]. Proteolytic cleavage of PARP activated caspases and can be used to detect apoptosis [42]. TA-1 was found here to trigger an increase in the expression levels of cleaved-PARP, as well as caspases-3, -8 and, -9 dependent increases in apoptosis. Caspases are activated by proteolytic processing at sites that are themselves caspase consensus cleavage sites [43]. An intrinsic pathway was activated in Limonene treated LS174T colon cancer cells [44].

Some EOs and their components inhibit tumor cell survival through caspase activation, the induction of proapoptotic proteins, and the downregulation of antiapoptotic proteins [1]. Activating caspase-8 is associated with FLICE/caspase-8 inhibitory protein (c-FLIP) decreasing after treatment with teucrium essential oil, which also inhibited the cell survival of HCT-116 cells by downregulating the expression of cyclin D1, myeloid cell leukemia (Mcl-1), X-chromosome-linked IAP (XIAP), IAP-1, IAP-2, and B cell lymphoma extra large (BcL-xL) levels. Bcl-2 protein is downregulated by the action of TA-1 on the HCT-116 cancer cells in a dose- and time-dependent manner. The results obtained here agreed with other reports that hydrophobic fractions extracted from many species are able to change the expression levels of Bcl-2 and *Bax* genes in KB human oral epidermoid carcinoma cells [11].

Furthermore, apoptosis and proliferation are linked by cell cycle regulators [45]. TA-1 induces apoptosis by increasing the tumor suppressor *p53* as a G1 regulator. p53 activates specific transcriptional targets that control cell cycle arrest, DNA repair, angiogenesis, autophagy, metabolism, migration, aging, senescence, and apoptosis [46]. Without a functional *p53* gene, cells lack the DNA-damage-sensing capability that would normally induce the apoptotic cascade [1]. Similar data were reported by Gupta *et al.* [47] in three colon cancer cell lines (HCT-116, Caco-2, and HT-29) that were inhibited by a triterpene (nimbolide) by repressing the expression of tumorigenic proteins.

STAT3 undergoes phosphorylation from serine residues [48]. In general, exposure to TA-1 is required to suppress the constitutive activation of STAT-3 (Figures 5(Aiv) and 7D) in HCT-116 cells. It can induce a decrease of phosphorylated signal transducers and the levels of activators of transcription protein STAT-3 (at Tyr^705^ and Ser^727^) in human colon cancer cells in a dose- and time-dependent manner. This data is supported by another report showing the ability of STAT-3 to phosphorylate in HCT-116 cell lines [49]. STAT-3 is overexpressed in several tumor cells, including cholangiocarcinoma, breast cancer, prostate cancer, head and neck squamous cell carcinoma, lymphomas and leukemia, brain tumors, gastric cancer, esophageal cancer, ovarian cancer, nasopharyngeal cancer, and pancreatic cancer [27].

In addition, Teucrium ethereal oil was also tested to see whether it was able to express proteins involved in the angiogenic phenotypes of cancer cells on tumor invasion and the metastasis of human colon cancer cells *in vitro* by reducing CXC chemokine receptor 4 (CXCR4) levels and restoring vascular cell adhesion molecule 1 (VCAM-1) and matrix metalloproteinase (MMP-9) expression in a dose- and time-dependent manner, as compared with control cells. MMP9 and ICAM-1 participate in the proteolytic degradation of tissue barriers [1]. The inflammatory process is directly linked to the metastatic process [50], which depends on angiogenesis. The potent antiangiogenic effect of TA-1 investigated in HCT-116 was associated with significant inhibition of the protein expression level of anti–vascular endothelial growth factor (VEGF), thus suggesting that this drug is a potent suppressor of tumor invasion through its action on VEGF, which induces angiogenesis by binding to its receptor tyrosine kinase. Chidambara and co-workers [51] investigated the antiangiogenic effect of d-limonene with a preventative effect on colon cancer. Jung *et al.* [52] showed that epigallocatechin gallate (EGCG) attenuated VEGF production through the inhibition of ERK-1 and ERK-2 kinases in human colon cancer cells. c-myc as a cell cycle component is down-regulated by teucrium essential oil, and this inhibition leads to growth arrest.

TA-1 inhibited the proliferation, invasion, and metastasis of Panc28, Miapaca, and HL60 cancer cells by down-regulating Bcl-xL, XIAP, survivin, MMP-9, and CXCR4 expression (Figure 6A–6C). CXCR4, cooperate with chemokine receptor (CCR1 and/or CCR2) to promote metastasis-associated macrophage (MAM) accumulation and thereby metastatic tumor growth [53]. Supplementary Figure 3 shows a densitometry analysis of Western blots. Understanding structural and molecular mechanisms of anti-cancer agents can help in developing new and more potent drugs with fewer side effects [27]. Deb and co-workers [54] reported that thymol induced apoptosis in HL-60 cells *via* caspase-dependent and caspase-independent pathways. A recent finding about d-limonene and elemene colon cancer treatment was obtained from LS174T and Lovo cells with *akt* and telomerase activity inhibition, respectively, cell cycle arrest, and apoptosis [44, 55]. d-limonene induced apoptosis in HL-60 cells through the activation of caspase-8 [56]. In another report, bioactive compound, 7-*O*-pentyl quercetin (Q-7P), induced apoptosis in tumoral cells and massive ROS production [57].

LPS is a major structural component of the outer membrane of Gram-negative bacteria and is a potent inducer of inflammation through the production of various cytokines, growth factors and inflammatory mediators [58]. It plays an important role in occurrence, development and metastasis of tumors [59]. Our data suggest that TA-1 act as a potential inhibitor for LPS-induced inflammation and damage of colon cells *in vivo* by decreasing the length of mice colon and body weight loss.

This study provides evidence for the therapeutic potential of *Teucrium alopecurus* essential oil to be used for the treatment of cancer. Thus, it up-regulates proapoptotic proteins and down-regulates cell survival, proliferation, invasion, angiogenesis, and metastasis in HCT-116 cells. Hydrolates obtained from this species have low potential as anti-cancer agents, and this may be due to low amounts of terpenes and phenolic compounds contained therein. Further studies in animals and humans will be required to demonstrate the relevance of these results obtained *in vitro* to cancer treatment. Essential oil of *Teucrium* is a potential therapeutic target for LPS-induced colon inflammation.

### Reagents

Hydrophobic and hydrophilic fractions of *Teucrium alopecurus* leaves were characterized by gas chromatography-mass spectrometry (GC/MS). Stock solutions of oily (100 μg/mL) and water soluble fractions (500 μg/mL) were prepared in dimethyl sulfoxide (DMSO) and diluted as needed in a cell culture medium. Penicillin, streptomycin, Iscove's modified Dulbecco's medium (IMDM), Roswell Park Memorial Institute *medium* (RPMI)-640 medium, Dulbecco's modified Eagle's medium (DMEM)/F12 medium and fetal bovine serum (FBS) were obtained from Mediatech, Inc. (Herndon, VA, USA). Bovine serum albumin (BSA) was purchased from Atlanta Biologicals (Norcross, GA, USA). NaCl, Tris, sodium dodecyl sulphate (SDS), MTT, and glycine, LPS (*Escherichia coli* 055:B5), the β-actin, butyl-hydroxytoluene (BHT), ascorbic acide (1%), 2,2-diphényl-1-picrylhydrazyl (DPPH), ferric reducing antioxidant power (FRAP), potassium ferricyanide (K_3_Fe(CN)_6_) (1%), trichloro acetic acide (TCA) (10%), ferric Chloride (FeCl_3_) (0.1%), CFM (10 μg/μl), and ERTA (10 μg/μl) were purchased from Sigma–Aldrich (St. Louis, MO, USA). Antibodies against c-Myc, CXCR4, MMP-9, cIAP-1/2, Mcl-1, ICAM-1, Bcl-xL, cyclin D1, caspases-3, -8, and -9, PARP, Bcl-2, STAT-3, and pSTAT3 (Tyr705 and Ser727) were obtained from Santa Cruz Biotechnology (Santa Cruz, CA, USA); the β-actin antibody was obtained from Sigma-Aldrich (St. Louis, MO, USA); X-linked IAP and c-FLIP antibodies were obtained from BD Biosciences and Imgenex (San Diego, CA, USA), respectively. Antibodies against survivin were obtained from R&D Systems (Minneapolis, MN, USA); the VEGF antibody was purchased from NeoMarkers (Fremont, CA, USA); 5-FU (purity > 99%) was purchased from Sigma Chemical Co.; Butyl-hydroxytoluene (BHT), Ascorbic acide (1%), 2,2-diphényl-1-picrylhydrazyl (DPPH), ferric reducing antioxidant power (FRAP), Potassium ferricyanide (K_3_Fe(CN)_6_) (1%), Trichloro acetic acide (TCA) (10%), Ferric Chloride (FeCl_3_) (0.1%), CFM (10 μg/μl), and ERTA (10 μg/μl) were obtained from Sigma-Aldrich Chemicals Co. (St. Louis, MO, USA).

### Cell lines

The cell lines HCT-116, MiaPaca-2 (Human pancreatic carcinoma), Panc28, KBM-5, SCC4 (human head and neck cancer), MCF7, U266 (human multiple myeloma), HL-60 (human promyelocytic leukemia), RAW 264.7 (mouse monocyte macrophage) and HUVECs were obtained from the American Type Culture Collection (Manassas, VA, USA). Human colon cancer cells, human pancreatic carcinoma, pancreatic carcinoma, human head and neck cancer, and RAW cells were cultured in Dulbecco's modified Eagle's medium (DMEM) supplemented with 10% fetal bovine serum (FBS). HL-60 and U266 cells were cultured in RPMI-1640 medium with 10% FBS; KBM-5 cells were cultured in Iscove's modified Dulbecco's medium (IMDM) supplemented with 15% FBS; and MCF-7 cell lines were cultured in DMEM/F12 with 10% FBS and no antibiotics. HUVECs cells were cultured in DMEM containing 10% FBS. All culture media were supplemented with 100 U/mL penicillin and 100 μg/mL streptomycin.

### Tested bacteria

The bacteria used in this study including *Escherichia coli* (*E.coli*), *Staphylococcus aureus* (*S.aureus*), *Bacillus subtilis* (*B.subtilis*), and *Klebsiella pneumoniae* (*K.pneumoniae*) were obtained from the laboratory of microbiology, Gafsa Hospital, Tunisia.

### Analysis of oily and hydrophilic fractions of *Teucrium alopecurus* by gas chromatography-mass spectrometry

*Teucrium alopecurus* leaves were collected from Orbata Mountain, Gafsa, Tunisia. A voucher specimen was deposited in the National Institute of Agronomic of *Tunisia* (INAT) Herbarium in Tunisia under registration number 1122. Decanted essential oil was separated, and the hydrosol was collected from the Clevenger distiller apparatus. To profile Teucrium essential oil and hydrosol bioactive compounds, GC/MS analysis was used. Different fractions were analysed using gas chromatography coupled with mass spectrometry (GC-MS) on an Agilent 6890 gas chromatograph with an autosampler coupled with an Agilent 5973 Mass Selective *Detector* (MSD) detector (Agilent Technologies, Palo Alto, CA, USA) with an electron impact ionization of 70 eV. A Phenomenex ZB-5MSi capillary column (30 m × 0.25 mm, 0.5 μm film thickness; Agilent Technologies, Hewlett-Packard, CA, USA) was used at a temperature programmed to rise from 40 to 280°C at a rate of 5°C/min. Helium (99.999% purity) was used as a gas carrier, with a flow rate of 0.7 mL/min, a split ratio of 60:1, and a scan time and mass range of 1s and 50–550 *m*/*z*, respectively. EOs were identified by matching the mass spectra recorded with those stored in the Wiley 09 NIST 2011 mass spectral library of the GC/MS data system.

### Phenolic content and antioxidant activity

Phenolic and flavonoid content were determined based on the method of Singleton and Rossi [60] and Dewanto *et al.* [61], respectively.

To verify that the CFl can scavenge the DPPH radicals (DPPH.), the modified DPPH method was measured as described by Brand-Williams *et al.* [62], using ascorbic acid as standard solution.

The evaluation of the ferric reducing antioxidant power (FRAP) of TA-1 and TA-2 was assessed by potassium ferricyanide-ferric chloride method as previously described by Oyaizu [63] with slight modifications, using BHT as standard solution.

### Disc diffusion assay

The antibacterial activity of TA-1 was evaluated by disc diffusion assay as described by Vlietinck *et al.* [64].

### Cell viability assay

RAW 264.7 (10^5^ cells/well) were seeded onto 96- well plates and incubated with different concentrations of Teucrium essential oil (0, 0.5, 1, 5, or 10 pg/mL) and water soluble fractions of Teucrium (0, 30, 50, 70, or 100 μg/mL) dissolved in DMSO for 24, 48, or 72 h. MTT solution was added and cells were incubated for 2 h at 37°C. Absorbance was measured at 570 nm using an MRX Revelation 96-well multiscanner (Dynex Technologies, Chantilly, VA, USA).

### Antiproliferative effect of TA-1

The antiproliferative effect of TA-1 was assessed by MTT assay as previously described [65]. 5 × 10^2^ cells per well were incubated with different concentrations of Teucrium essential oil (0, 0.5, 1, 5, or 10 pg/mL) and water soluble fractions of Teucrium (0, 30, 50, 70, or 100 μg/mL) dissolved in DMSO for one, three, or five days in 96-well plates. Vehicle was considered as control. After treatment, the MTT solution was added to various tumor cells, which were incubated for 2 h at 37°C before adding the extraction buffer. The cells were incubated overnight at 37°C. Absorbance was measured at 570 nm using an MRX Revelation 96-well multiscanner (Dynex Technologies, Chantilly, VA, USA).

### Live/dead assay

Apoptosis was measured by Live/Dead assay, which determines the number of live and dead cells. After the treatment of various tumor cells (2 × 10^5^) with 0.0005, 0.001, 0.005, 0.01, or 0.05 pg/mL of TA-1 and 3.75, 5, 10, or 15 μg/mL of TA-2, the treated cells and untreated cells were stained with a a green fluorescent dye, a nonfluorescent polyanionic dye, calcein-AM, which is retained within live cells, and Ethidium homodimer dye, a red fluorescent that can enter cells through damaged membranes, and the cells were incubated for 30 min at 37°C. The cells were analyzed under a fluorescence microscope (Labophot-2; Nikon, Melville, NY, USA). After counting the live and dead cells, the percentage of cells in apoptosis in each sample was calculated.

### Clonogenic assay

To determine tumor growth, a clonogenic assay was done. The cells were treated with different concentrations of TA-1 and TA-2, and then the treated and untreated cells were seeded in Petri dishes, allowed to form colonies for two weeks, and then stained, as indicated by Takada and Aggarwal [65].

### Production of ROS levels in HCT116

Colon cancer cells (1 × 10^6^ cells) cells were labeled with dichlorodihydrofluorescein diacetate (DCF-DA) (20 μM) and exposed to different concentrations of oily and soluble fractions of Teucrium species for 1 h. The increase in fluorescence resulting from the oxidation of DCF-DA to DCF was measured by fluorescence-activated cell sorting (FACS). The mean fluorescence intensity at 530 nm was calculated. The results were collected from at least 10,000 cells at a flow rate of 250–300 cells·s^−1^ [66].

### Western blot analysis

Western blotting is used for the detection and analysis of proteins based on their ability to bend to specific antibodies. Cell extracts (treated and untreated cancer cells, normal cells) were prepared in lysis buffer (20 mM, Tris (pH 7.4), 250 Mm NaCl, 2 Mm ethylenediaminetetraacetic acid (EDTA) (pH 8.0), 0.1% triton X-100, 0.01 μg/mL aprotinin, 0.005 μg/mL leupeptin, 0.4 M phenylmethylsulfonyl fluoride, and 4 Mm Na_3_VO_4_). To remove insoluble materials, we centrifuged lysates at 1400 rpm for 10 min. The supernatant was collected and kept at −80°C. Thirty μg of lysates were resolved by 10% SDS-PAGE and the proteins were then transferred to nitrocellulose membranes where they were bound, forming the blot. After that, the membranes were probed overnight with relevant primary antibodies at 4°C. After the blotting process, the proteins were exposed to a thin surface layer for primary antibody detection. The dilution factors of antibodies are: c-Myc (1:2000), CXCR4 (1:5000), MMP-9 (1:1000), cIAP-1/2 (1:2000), Mcl-1 (1:3000), ICAM-1 (1:2000), Bcl-xL (1:2000), cyclin D1 (1:3000), caspase-3 (1:2000), -8 (1:2000) and -9 (1:2000), PARP (1:5000), Bcl-2 (1:3000), STAT-3 (1:3000), pSTAT3 (Tyr705 and Ser727) (1:2000), β-actin (1:10,000), XIAP (1:3000), c-FLIP (1:3000), survivin (1:2000), VEGF (1:1000), *p53* (1:3000), and TRAF1 (1:2000).

Then, blots, blocked with 5% nonfat milk, were incubated with peroxidase-conjugated secondary antibodies (goat anti-mouse or goat anti-rabbit) (1:5000) diluted in 5% skimmed milk for 1 h, and signals were detected by enhanced chemiluminescence reagent (GE Healthcare, Piscataway, NJ, USA). Vehicle was considered as control. The bands obtained were quantitated using NIH imaging software (Available online: https://imagej.nih.gov/ij/download.html).

### Acute toxicity test

To evaluate the cytoxicity effect of TA-1, four groups of mice (6 mice per group), obtained from the experimental animal house, sfax, Tunisia, were given standard pellets and tap water and treated with increasing doses of the essential oil (0-10-20-30 μg/kg). The essential oil administration was prepared by diluting the stock solution of TA-1 by mixing it with 2% Tween 80. After treatment, the animals were kept under observation daily for any behavioral changes, mortality or consumption of food and water. After 7 days, the mice were euthanized, and the liver and kidney tissues were used for macroscopically and histological overview.

### *In vivo* antitumour activity

#### Animal studies

A total of 48 Swiss albino mice (20 ± 1.00 g) were obtained from the Animal laboratory of Sfax, Tunisia. Mice were maintained for a week in a propylene cage at 20 ± 25°C with relative humidity of 55% ± 10% under a cycle of 12 h light/dark. Animals were allowed *ad libitum* to access to tap water and food pellets.

#### Experimental procedures

The essential oil used in this report was dissolved in 2% Tween 80 and given to mice intragastrically once a day for 7 consecutive days. At the beginning of the experiment, the mice were divided into eight groups (6 mice per group) as follows: Group 1 : mice treated by intragastrically administration of vehicle (2% Tween 80 in distilled water); Group 2: served as positive (tumor) control, mice treated by intragastrically administration of LPS (*Escherichia coli* 055:B5) (10 μg/ml); Group 3: animals treated by intragastrically administration of the essential oil (10 μg/kg/day); Group 4: mice treated by intragastrically administration of the essential oil (20 μg/kg/day). The treatments were started daily, one hours before LPS treatment; Group 5: served as comparator control, mice treated by intragastrically administration of 5-fluorouracil (5-FU) (20 mg/kg/day). Group 6: TA-1 LD combined with LPS (TA-1 LD + LPS) (10 μg/kg, body weight/day and 10 μg/ml, respectively); Group 7: TA-1 HD combined with LPS (TA-1 HD + LPS) (20 μg/kg, body weight/day and 10 μg/ml, respectively); Group 8: 5-Fu combined with LPS (5-Fu + LPS) (20 mg/kg, body weight/day and 10 μg/ml, respectively). All the treatments were given orally once daily for 7 days. After 7 days, the mice were euthanized; lung, spleen and colonic tissues were collected to be analyzed macroscopically and microscopically by Hematoxylin & Eosin staining. The experiments were carried out according to Guide for the Care and Use of laboratory Animals approved by the Animal Ethics Committee.

### Statistical analysis

Different parameters were monitored in normal and treated cells. Experiments were repeated a minimum of three times. The results were expressed as mean ± SD. Differences between groups were compared by a one-way analysis of variance (ANOVA). A value of *p* < 0.05 was considered statistically significant.

## SUPPLEMENTARY MATERIALS FIGURES AND TABLE





## Abbreviations

MTT: Tetrazolium salt 3-(4-5-dimethylthiozol-2-yl)2-5-diphenyl-tetrazolium bromide
VEGF: Anti–vascular endothelial growth factor
Bcl-xL: B-cell leukemia protein xL
cIAP: Cellular inhibitor of apoptosis protein
PARP: Poly-adenosine diphosphate ribose polymerase
c-FLIP: Cellular FLICE-inhibitory protein
XIAP: X-linked IAP
ICAM-1: Intercellular adhesion molecule-1
CXCR4: CXC chemokine receptor-4
FLICE: FADD-like IL-1β-converting enzyme
ROS: Reactive Oxygen Species
STAT3: Signal transducer and activator of transcription 3
TRAF: TNF receptor-associated factor
