# Supplementary material for: Terpenes from essential oils and hydrolate of Teucrium alopecurus triggered apoptotic events dependent on caspases activation and PARP cleavage in human colon cancer cells through decreased protein expressions
Source: Oncotarget. 2018 Aug 17;9(64):32305–20. doi: 10.18632/oncotarget.25955 (PMC6122345; doi:10.18632/oncotarget.25955)
Supplement: Supplementary file 1 [file oncotarget-09-32305-s001.pdf]

Terpenes from essential oils and hydrolate of *Teucrium alopecurus* triggered apoptotic events dependent on caspases activation and PARP cleavage in human colon cancer cells through decreased protein expressions

SUPPLEMENTARY MATERIALS

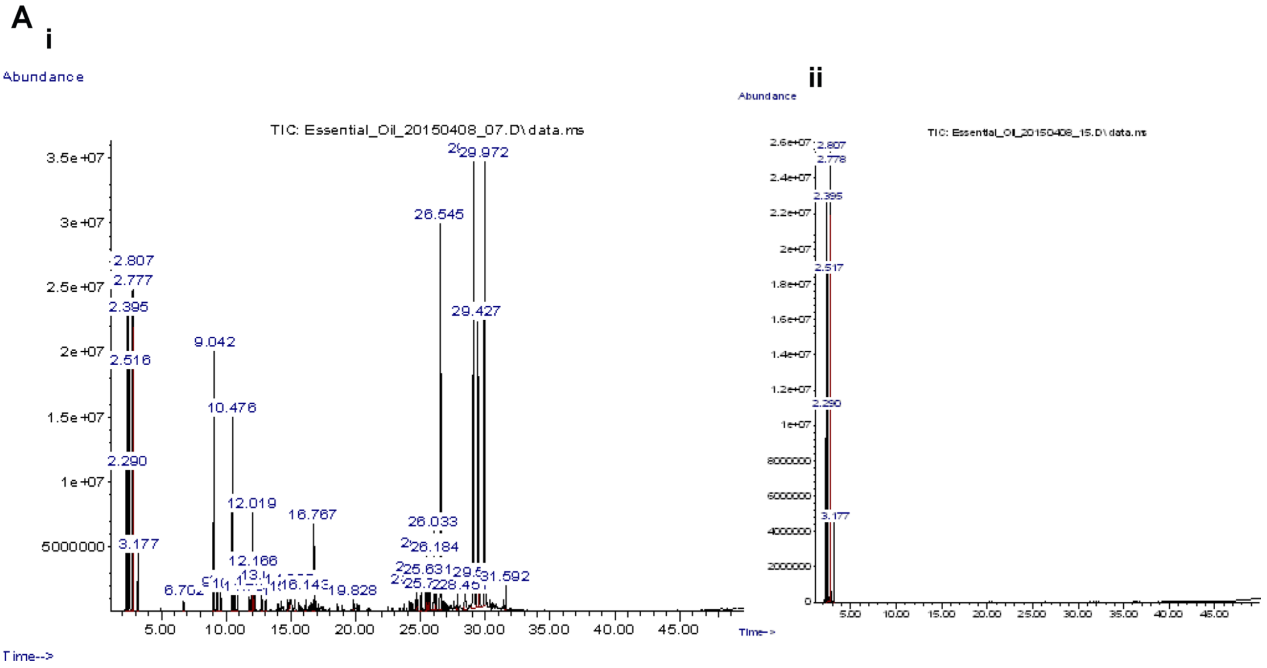

Supplementary Figure 1: Essential oil chromatogram of volatile compounds in the aerial part of Hydrophobic (TA-1) (**i**) and Hydrophilic (TA-2) (**ii**) fractions of Teucrium alopecurus.

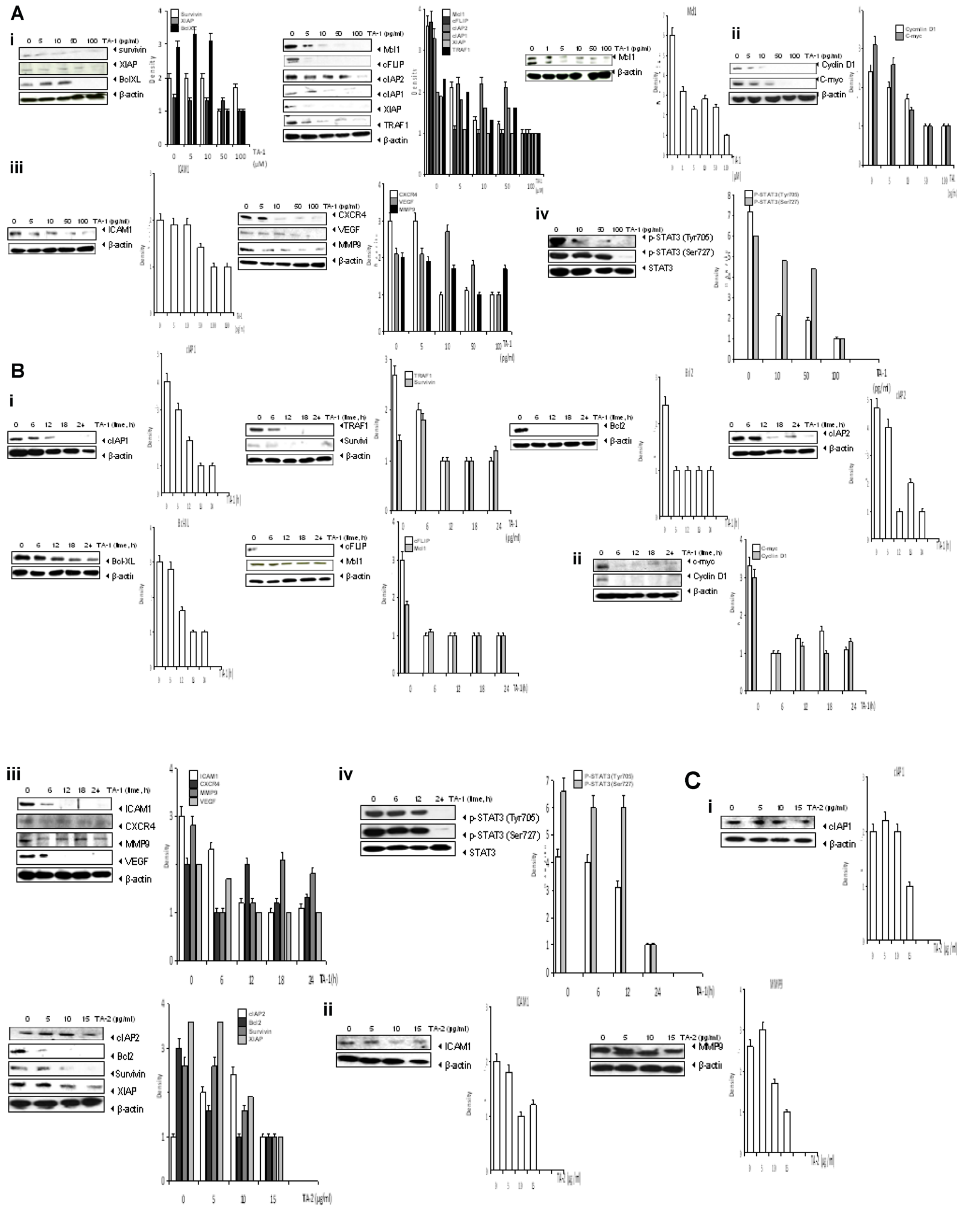

Supplementary Figure 2: Western blotting and relative densitometric analyses of suppression of antiapoptotic (**Ai**, **Bi**), proliferative and metastatic (**Aii**, **Aiii**, **Bii**, **Biii**) gene products and inhibition of STAT3 phosphorylation (**Aiv**, **Biv**) cellular levels by TA-1 essential oil in HCT116 cell lines at different concentrations and time poi nts. Western blotting and relative densitometric analyses of suppression of antiapoptotic (**Ci**) and metastatic (**Cii**) gene products cellular levels by TA-2 hydrolate in HCT116 cell lines different concentrations. Blots have been done three times.

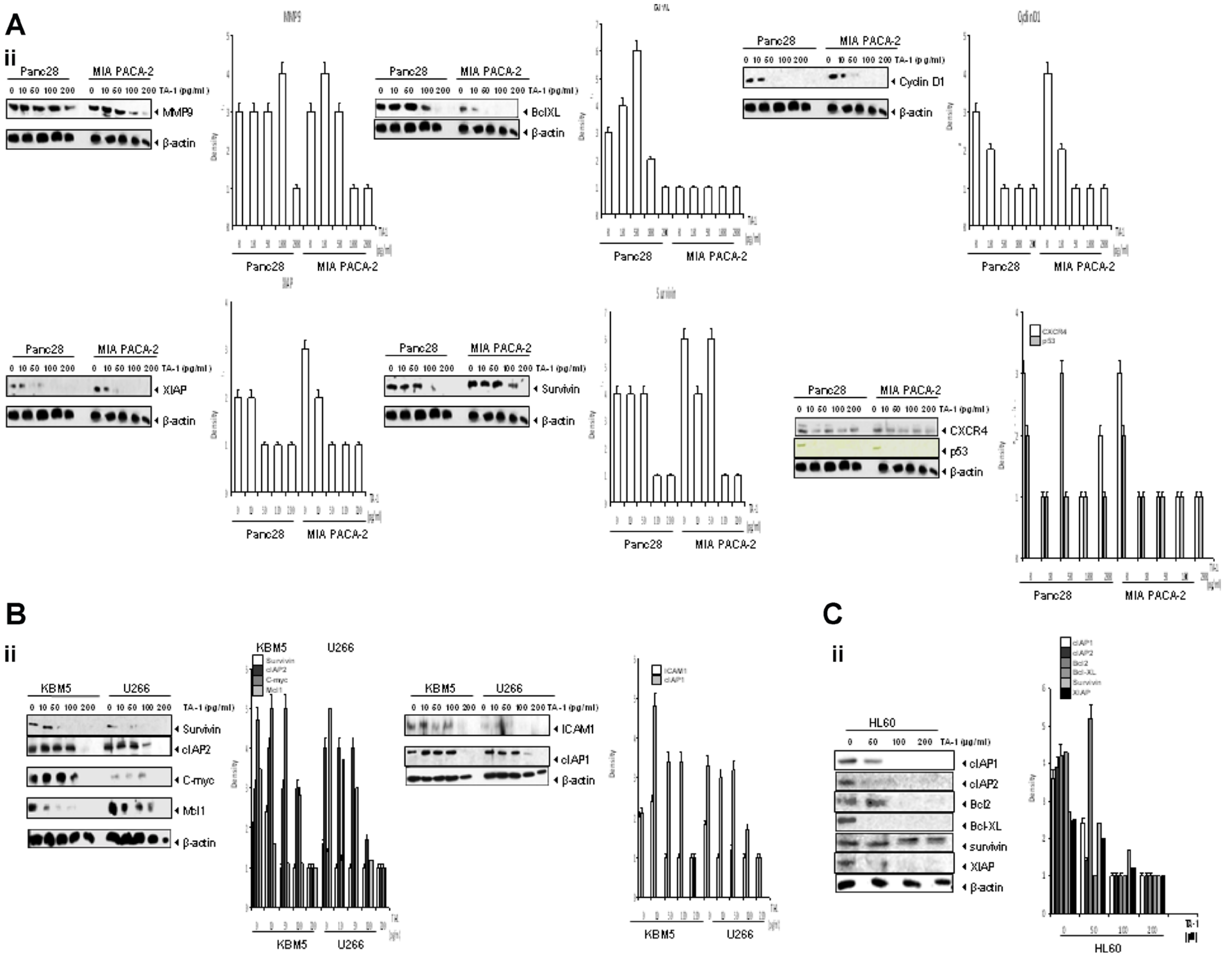

Supplementary Figure 3: Graphs show densitometry analysis of Western blots for suppression of antiapoptotic, proliferative and metastatic (**Aii**, **Bii**, **Cii**) gene products cellular levels by TA-1 essential oil in both Panc28- and MIA PACA-2 (**A**) cell lines, KBM5 and U266 cells (**B**) and HL60 cells (**C**) at different concentrations for 24 h, normalized to β-actin expression loading control. Blots have been done three times.

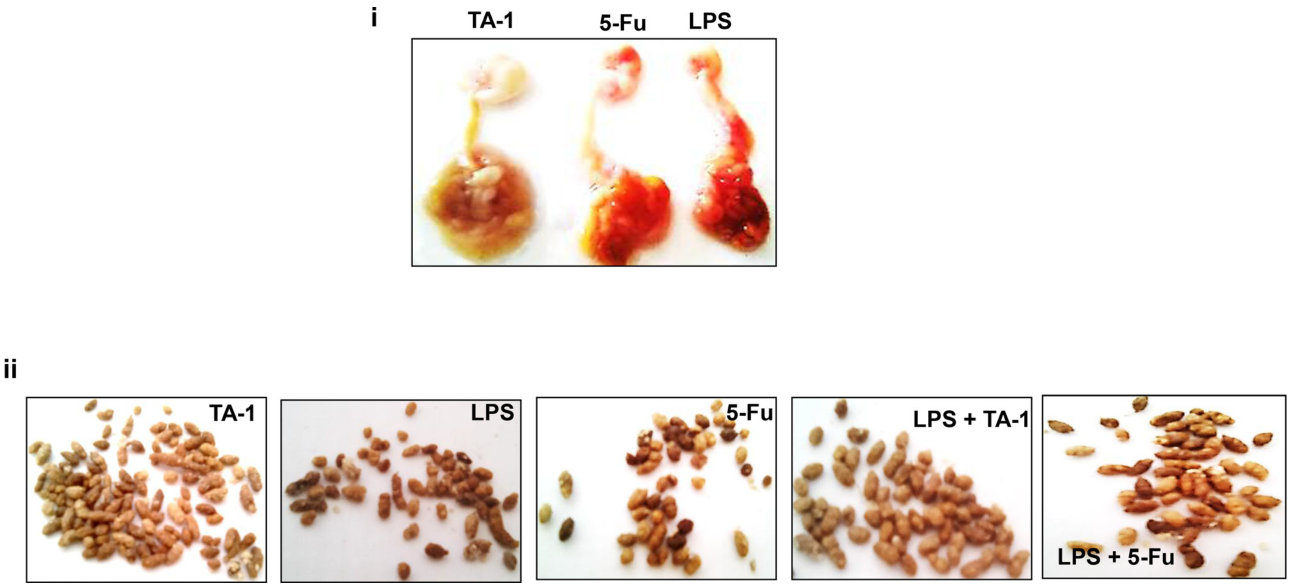

Supplementary Figure 4: Macroscopic view of mice feces (**i**) and digestive system (**ii**) of different groups.

Supplementary Table 1: Chemical composition of aerial parts of Teucrium alopecurus essential oil, cultivated in Tunisia. See Supplementary_Table_1
