# Supplementary material for: Terpenes from essential oils and hydrolate of Teucrium alopecurus triggered apoptotic events dependent on caspases activation and PARP cleavage in human colon cancer cells through decreased protein expressions
Source: Oncotarget. 2018 Aug 17;9(64):32305–20. doi: 10.18632/oncotarget.25955 (PMC6122345; doi:10.18632/oncotarget.25955)
Supplement: Supplementary file 2 [file oncotarget-09-32305-s002.docx]

**Supplementary Table 1:** Chemical composition of aerial parts of *Teucrium alopecurus* essential oil, cultivated in Tunisia

| **NO.** | **RI^a^** | **Components^b^** | **Formula** | **Pic Area (%)** **TA-1** | **Identification** |
| --- | --- | --- | --- | --- | --- |
| 1 | 939 | Pentane, 2-methyl | C_6_H_14_ | 1.46 | GC-MS |
| 2 | 946 | 1-Pentene | C_5_H_10_ | tr **^c^** | GC-MS |
| 3 | 948 | Pentane, 3-methyl | C_6_H_14_ | 6.12 | GC-MS |
| 4 | 952 | n-Hexane | C_6_H_14_ | tr | GC-MS -RT |
| 5 | 955 | Cyclopentane, methyl | C_6_H_12_ | 2.47 | GC-MS |
| 6 | 958 | 1-Butene, 3,3-dimethyl- | C_6_H_12_ | tr | GC-MS |
| 7 | 962 | Cyclohexane | C_6_H_12_ | 0.67 | GC-MS –RI |
| 8 | 965 | Borane | BH_3_ | 0.19 | GC-MS –RI |
| 9 | 967 | Bicyclo[3.1.0]hex-2-ene, 4-methyl-1-(1-methylethyl)- | C_10_H_16_ | 5.12 | GC-MS |
| 10 | 968 | (1R)-2,6,6-Trimethylbicyclo[3.1.1] hept-2-ene | C_10_H_16_ | 0.44 | GC-MS |
| 11 | 970 | (1S)-2,6,6-Trimethylbicyclo[3.1.1] hept-2-ene | C_10_H_16_ | 0.44 | GC-MS |
| 12 | 972 | Benzene, butyl | C_10_H_14_ | tr | GC-MS |
| 13 | 976 | β-Phellandrene | C_10_H_16_ | 3.88 | GC-MS -CAS# |
| 14 | 979 | β-Pinene | C_10_H_16_ | 0.48 | GC-MS |
| 15 | 993 | β-Myrcene | C_10_H_16_ | 0.35 | GC-MS–CAS# |
| 16 | 1022 | (+)-2-Carene | C_10_H_16_ | 0.31 | GC-MS |
| 17 | 1026 | o-Cymene | C_10_H_14_ | 2.00 | GC-MS |
| 18 | 1028 | p-Cymene | C_10_H_14_ | 2.00 | GC-MS |
| 19 | 1037 | trans-3(10)-Caren-2-ol | C_10_H_16_O | 0.77 | GC-MS |
| 20 | 1041 | d-Limonene | C_10_H_16_ | 0.89 | GC-MS |
| 21 | 1069 | Ethanone, 1-(1,4-dimethyl-3-cyclohexen-1-yl)- | C_10_H_16_O | 0.46 | GC-MS |
| 22 | 1071 | γ-Terpinene | C_10_H_16_ | 0.53 | GC-MS |
| 23 | 1132 | 2-(5-Methyl-furan-2-yl)-propionaldehyde | C_8_H_10_O_2_ | 0.24 | GC-MS |
| 24 | 1177 | 4-Acetyl-1-methylcyclohexene | C_9_H_14_O | 0.35 | GC-MS |
| 25 | 1185 | Methyl ethyl cyclopentene | C_8_H_14_ | 0.43 | GC-MS |
| 26 | 1193 | Terpinen-4-ol | C_10_H_16_O | 1.88 | GC-MS |
| 27 | 1293 | Thymol | C_10_H_14_O | 0.24 | GC-MS |

**-Continued-**

| **NO.** | **RI^a^** | **Components^b^** | **Formula** | **Pic Area (%)** **TA-1** | **Identification** |
| --- | --- | --- | --- | --- | --- |
| 28 | 1347 | (+)-(E)-Limonene oxide | C_10_H_16_O | 0.37 | GC-MS |
| 29 | 1420 | 4,11-selinadiene | C_15_H_24_ | 0.93 | GC-MS |
| 30 | 1484 | β-Selinene | C_15_H_24_ | 1.31 | GC-MS |
| 31 | 1488 | cis-α-Bisabolene | C_15_H_24_ | 0.46 | GC-MS |
| 32 | 1491 | α-Selinene | C_15_H_24_ | 0.78 | GC-MS |
| 33 | 1505 | β-Bisabolene | C_15_H_24_ | 0.34 | GC-MS |
| 34 | 1513 | γ-Cadinene | C_15_H_24_ | 1.78 | GC-MS |
| 35 | 1523 | δ-Cadinene | C_15_H_24_ | 1.33 | GC-MS |
| 36 | 1554 | Humulene | C_15_H_24_ | tr | GC-MS |
| 37 | 1584 | β-Caryophyllene oxide | C_15_H_24_O | 0.37 | GC-MS |
| 38 | 1593 | 3-Cyclohexen-1-carboxaldehyde, 3,4 dimethyl- | C_9_H_14_O | 0.73 | GC-MS |
| 39 | 1607 | α-humulene epoxide | C_15_H_24_O | 0.73 | GC-MS |
| 40 | 1616 | Pentylidenecyclohexane | C_11_H_20_ | tr | GC-MS |
| 41 | 1634 | (+)-epi-Bicyclosesquiphellandrene | C_15_H_24_ | 15.40 | GC-MS |
| 42 | 1637 | Bicyclo[4.4.0]dec-1-ene, 2-isopropyl-5-methyl-9-methylene- | C_15_H_24_ | Tr | GC-MS |
| 43 | 1640 | α-Cadinol | C_15_H_26_O | 8.52 | GC-MS |
| 44 | 1641 | Tau-Muurolol | C_15_H_26_ | tr **^c^** | GC-MS |
| 45 | 1649 | Guaia-3,9-diene | C_15_H_24_ | tr | GC-MS |
| 46 | 1683 | α-Bisabolol | C_15_H_26_OH | 16.16 | GC-MS |
| 47 | 1707 | α-cyperone | C_15_H_22_O | 0.53 | GC-MS |
| 48 | 1734 | 2(1H)Naphthalenone, 3,5,6,7,8,8a-hexahydro-4,8a-dimethyl-6-(1-methylethenyl)- | C_15_H_22_O | 0.53 | GC-MS |
| **Chemical classes** | | | | | |
|  | | Monoterpene Hydrocarbons | 12.44 | | |
|  |  | Oxygenated monoterpenes | 1.50 | | |
|  |  | Sesquiterpene Hydrocarbons | 22.33 | | |
|  |  | Oxygenated Sesquiterpenes | 1.10 | | |
|  |  | Oxygenated diterpenes | 0 | | |
|  |  | Others | 44.52 | | |
|  |  | Total identified % | 81.99 | | |
|  |  | Oil Yield (%) | 2.00 | | |

(a) Retention Index relative to C_5_-C_15_ n-alkanes on the phenomenex ZB-5MSi capillary column; (b) Compounds listed in order of their RI, (c) tr = trace (<0.05%)
